# Development of an electronic conversation aid to support shared decision making for children with acute otitis media

**DOI:** 10.1093/jamiaopen/ooab024

**Published:** 2021-04-19

**Authors:** Jana L Anderson, Lucas Oliveira J e Silva, Juan P Brito, Ian G Hargraves, Erik P Hess

**Affiliations:** 1 Department of Emergency Medicine, Mayo Clinic, Rochester, Minnesota, USA; 2 Department of Internal Medicine, Division of Endocrinology, Diabetes, Metabolism, and Nutrition, Mayo Clinic, Rochester, Minnesota, USA; 3 Knowledge and Evaluation Research Unit, Mayo Clinic, Rochester, Minnesota, USA; 4 Department of Emergency Medicine, Vanderbilt University, Nashville, Tennessee, USA

**Keywords:** otitis media, child, acute pain, decision making, shared, clinical decision making

## Abstract

**Objective:**

The overuse of antibiotics for acute otitis media (AOM) in children is a healthcare quality issue in part arising from conflicting parent and physician understanding of the risks and benefits of antibiotics for AOM. Our objective was to develop a conversation aid that supports shared decision making (SDM) with parents of children who are diagnosed with non-severe AOM in the acute care setting.

**Materials and Methods:**

We developed a web-based encounter tool following a human-centered design approach that includes active collaboration with parents, clinicians, and designers using literature review, observations of clinical encounters, parental and clinician surveys, and interviews. Insights from these processes informed the iterative creation of prototypes that were reviewed and field-tested in patient encounters.

**Results:**

The ear pain conversation aid includes five sections: (1) A home page that opens the discussion on the etiologies of AOM; (2) the various options available for AOM management; (3) a pictograph of the impact of antibiotic therapy on pain control; (4) a pictograph of complication rates with and without antibiotics; and (5) a summary page on management choices. This open-access, web-based tool is located at www.earpaindecisionaid.org.

**Conclusions:**

We collaboratively developed an evidence-based conversation aid to facilitate SDM for AOM. This decision aid has the potential to improve parental medical knowledge of AOM, physician/parent communication, and possibly decrease the overuse of antibiotics for this condition.

**LAY SUMMARY**

The overuse of antibiotics for acute otitis media (AOM) in children is a healthcare quality issue in part arising from conflicting parent and physician understanding of the risks and benefits of antibiotics for AOM. Parents frequently view antibiotics as a way to decrease immediate ear pain, where antibiotics have been found to have minimal effect in the first 48 hours on ear pain. We developed a web-based encounter tool to facilitate conversations between clinicians and parents when making management decisions for children with non-severe AOM. This tool was specifically developed for clinical scenarios with children diagnosed with non-severe AOM in which initial observation is a reasonable option.

## INTRODUCTION

Acute otitis media (AOM) is often defined as the rapid onset of signs and symptoms of inflammation in the middle ear. Distinguishing between viral and bacterial etiologies is challenging and invasive; none the less, allowing the infection to take a natural course without antibiotics is increasingly being recommended in immunocompetent children with mild symptoms.^1^ The American Academy of Pediatrics (AAP) and American Academy of Family Practice (AAFP) have written a joint guideline on the management for AOM which emphasizes that in certain pediatric populations of non-severe disease, observation with close follow-up is reasonable.^1^ Specifically, there are two patient populations addressed by these guidelines where initial observation with delayed antibiotic therapy is recommended as opposed to immediate treatment: (1) non-severe unilateral AOM in children 6 months to 23 months of age and (2) non-severe AOM in children 24 months or older. Non-severe is defined as AOM with mild otalgia for less than 48 hours and temperature less than 39°C. Regardless of guidelines recommendations, families are often reluctant to forgo antibiotics due to the perceived notion that antibiotics are required to resolve the infection despite the high probabilities of viral etiologies and self-limited illness.^2^ Similarly, practitioners are apprehensive about withholding antibiotics because of patient and parent dissatisfaction and concern for complications.

Shared decision making (SDM) is an approach that supports patients and clinicians in finding a path forward when there is uncertainty about which treatment option is best or when there is conflict about what to do. Shared decision making can be facilitated by the use of encounter conversation aids, sometimes referred to as decision aids.^3^ These tools, when used by clinicians and patients together, have been shown to be effective in creating the space for evidence-informed conversations. Encounter conversation aids improve knowledge about options, calibrate understanding of prognosis about the condition, and frame expectations about the impact of treatment options. In fact, for certain clinical scenarios in the emergency department (ED) and acute care settings, the use of decision aids as an instrument for SDM has demonstrated beneficial effects on patient knowledge.^4^^,^^5^ For example, in a multicenter pragmatic randomized trial, low-risk adult patients with a primary complaint of chest pain who were assigned to receive a SDM intervention facilitated by a decision aid had significantly greater knowledge of their risk for acute coronary syndrome and options for care when compared with the usual care arm.^5^ The evidence on the use of decision aids in adult SDM is extensive,^6^ but high-quality evidence is sparse in the pediatric literature. Although of limited study quality, previously tested SDM interventions in children seem to be associated with increased parent knowledge and decreased decisional conflicts.^7^ Nevertheless, whether this translates to overall better clinically important outcomes is yet to be further investigated in specific clinical pediatric scenarios.

The decision to prescribe antibiotics for pediatric AOM might be made without meaningful engagement with parents. The uncertainty and potential harms associated with antibiotic use create a scenario in which parents’ values and preferences should be considered.^8^ Besides prescribing pain control medications, the decision in question is broken down into two possible management options: immediate treatment with antibiotics versus observation with initiation of antibiotic therapy if the signs and symptoms worsen or fail to improve after 48 to 72 hours. In the latter scenario, a prescription is often provided to the parent to fill if needed.^1^^,^^9^ Despite a decision to observe the child for 48 to 72 hours prior to the initiation of antibiotics, it is unclear how frequently parents fill the prescription for antibiotics and forego the advice to wait and watch. The use of a decision aid has potential to support the conversation between the parent and physician and develop a common understanding of the current evidence for wait and watch prescriptions. In this report, we describe the development of an encounter tool to support SDM with parents of children with a diagnosis of non-severe AOM where initial observation with delayed antibiotic therapy is as reasonable as immediate treatment. Importantly, this tool was not intended to be used in more complex patient populations such as those with severe AOM, previous or current use of ear tubes, or multiple recurrent AOM.

## METHODS

### The development approach

**Figure 1.**
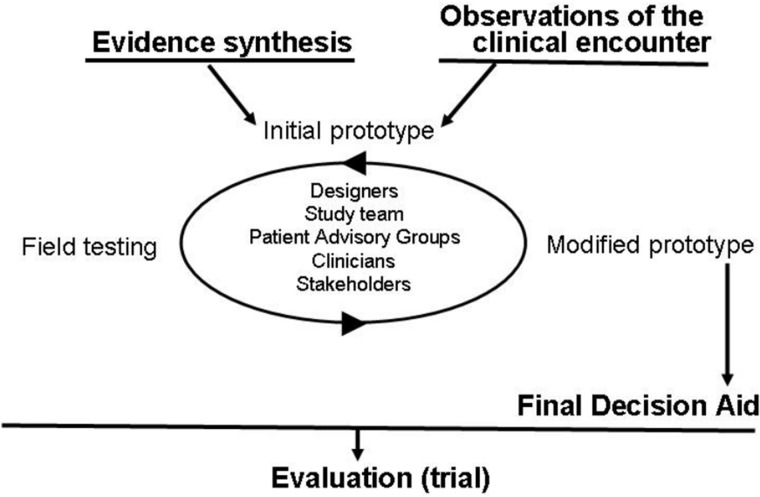
General approach for the developmental process of the ear pain decision aid.

We used a practice-based, patient-centered approach to develop the ear pain decision aid (Figure 1). This process included six main elements: (1) surveys of patients and clinicians regarding AOM and its treatment; (2) interviews with parents; (3) synthesis of clinical evidence regarding the risks and benefits of each management option; (4) observations of real-time clinical encounters involving clinicians and parents of children with AOM; (5) prototyping of the conversation aid through a series of iterations and field testing in the same context as the initial observations; and (6) solicitation of parent, clinician, and patient advisory board feedback on prototypes. This approach to conversation aid development and refinement is based on human-centered design and participatory action research methods developed and validated by the Knowledge and Evaluation Research Unit at the Mayo Clinic.^10^ The design process was also guided by the International Patient Decision Aid Standards (IPDAS) criteria.^11^ The core development team was composed of a pediatric emergency medicine clinician, a parent of a child with AOM in the past, and a designer. The core team incorporated literature, surveys, patient encounters, and patient and physician viewpoints to develop the final version of the conversation aid.

This process enabled the development team to learn more about what is necessary to make SDM possible for children with AOM in the acute care setting. In each step, stakeholders were engaged to review the findings and provide feedback to improve the tool. This approach has been similarly used to develop other conversation aids^4^^,^^12–14^ and involves active partnership with end users.

### Interviews and surveys with parents and clinicians

In the early phases of the development process, a convenience sample of 21 parents of children who either were being seen in the ED for ear pain or had a history of AOM within the last 2 years were surveyed, and 15 parents were informally interviewed by the lead author (pediatric emergency physician) in order to complement survey responses. The survey used is available in Supplementary Appendix S1. Descriptive analysis of the surveys was performed. The majority of children (*n* = 13/21, 61.9%) were between 6 months and 2 years of age at the time of AOM diagnosis and one (4.8%) was less than 6 months. Once the diagnosis of AOM was made, pain control was the leading concern of parents followed by the desire for antibiotics. A significant proportion (*n* = 8/21, 38%) of parents felt that antibiotics were either “always” required or needed “most of the time” for an ear infection. Additional data from this survey is provided in Table 1. The interviews’ predominant themes resonated with the survey responses including the importance of pain control and the common desire for antibiotics.

**Table 1.** Results from the survey of 21 parents in the early period of the development phase

Survey questions	Results
How old was your child when he/she was diagnosed with their most recent ear infection?	<6 months: 1/21 (4.8%) 6 months to 2 years: 13/21 (61.9%) 2 to 5 years: 5/21 (23.8%) >5 years: 2/21 (9.5%)
How long ago was your child’s most recent ear infection?	<6 months: 10/21 (47.6%) 6 months to 1 year: 7/21 (33.3%) 1 to 2 years: 3/21 (14.3%) >2 years: 1/21 (4.8%)
How many ear infections has your child had in the past year?	Mean 1.5 (SD 1.1) Median 1 (range 0 to 4)
Which symptom made you seek care for the ear infection? Number the most important (1) to least important (8)	10 survey responses had a complete report with the grading of importance for the symptoms. Fever was graded as the most important symptom in 5/10 (50.0%), followed by pain in 2/10 (2/10, 20.0%), pulling ear in 2/10 (20.0%), and crying in 1/10 (10.0%)
What did you hope most to gain from your last visit for the ear infection? Number the most important (1) to the least important (5)	15 survey responses had a complete report with the grading of importance. Diagnosis was graded as the most important factor in 9/15 (60.0%), followed by pain control in 4/15 (26.7%), and antibiotics in 2/15 (13.3%)
What worried you most about your child’s last ear infection? Number the most important (1) to the least important (5)	17 survey responses had a complete report with the grading of importance Pain was graded as the most important concern in 7/17 (41.2%), followed by fever in 4/17 (23.5%), ear drum rupturing in 2/17 (11.8%), difficulty sleeping in 2/17 (11.8%), and infection spreading in 1/17 (5.9%)
What was your child treated with?	Tylenol or ibuprofen: 13/20 (65.0%) Antibiotics: 16/20 (80.0%) Ear drops: 5/20 (25.0%) Nothing: 0/20 (0.0%) I don’t remember: 1/20 (5.0%)
How involved were you in the decision to treat your child?	Extremely: 10/21 (47.6%) Very: 5/21 (23.8%) Moderately: 5/21 (23.8%) Slightly: 1/21 (4.8%) Not at all: 0/21 (0.0%)
How long did it take for your child to feel better?	1 day: 2/18 (11.1%) 2–3 days: 12/18 (66.7%) 4–7 days: 1/18 (5.6%) 1 week: 3/18 (16.7%)
Circle the response that fits best your idea of treating ear infections. “Antibiotics are needed to treat ear infections…”	Always: 4/21 (19.0%) Most of the times: 4/21 (19.0%) Sometimes: 12/21 (57.1%) Not very often: 1/21 (4.8%) Never: 0/21 (0.0%) Not sure: 0/21 (0.0%)
Did your child have any problems after the ear infection was diagnosed?	No problems: 11/18 (61.1%) Repeat ear infection: 4/18 (22.2%) Mastoiditis: 0/18 (0.0%) Meningitis: 0/18 (0.0%) Cerebral venous thrombosis: 0/18 (0.0%) Other: 3/21 (14.3%)—1 reported need of “stronger antibiotic” and 2 reported “allergy”
Which of these five statements best describes how you prefer to make medical decisions?	“I make decisions about my child’s health care”: 1/20 (5.0%) “I make decisions about my child’s health care after seriously considering my clinician’s opinion”: 9* /20 (45.0%) “My clinician and I share responsibility for making decisions about my child’s health care”: 9/20 (45.0%) “My clinician makes decisions about my child’s health care, but seriously considers my opinion”: 2/20 (10.0%) “My clinician makes decisions about my child’s health care”: 0/20 (0.0%)

† The completeness of the survey was heterogeneous across the different questions, and, for this reason, the denominator may be different than 21 in some of the questions. This was a self-administered survey in a paper format. More details for each question included are available in Supplementary Appendix S1.

* One parent marked both the first and the second options rather than choosing only one.

Informal physician input was also gathered at local and national forums. Physicians were generally open to the observation approach (i.e. “wait and see”) for the majority of their patients depending on age. The age cutoffs were those described by the AAP clinical practice guideline involving the target population of this conversation aid: (1) non-severe unilateral AOM in children 6 months to 23 months of age and (2) non-severe AOM in children 24 months or older. The majority of clinicians felt that they followed the AAP/AAFP guidelines on AOM.^1^

### Evidence synthesis

Systematic reviews are tools to synthesize evidence. They are traditionally placed at the top of the evidence-based medicine pyramid and can be seen as a lens through which evidence is viewed.^15^ We chose a 2015 Cochrane systematic review^9^ as the primary evidence source regarding antibiotic use for initial development of the conversation aid. In addition, the joint guideline from the AAP/AAFP, updated in 2013, was also used as a resource for evidence aggregation.^1^ Lastly, a summary of potential complications of AOM were updated from another review of the literature.^16^

### Observations of real-time clinical encounters

Prior to developing a prototype, we observed and recorded conversations between patients and clinicians regarding AOM treatment in an ED setting. Five pediatric emergency physicians participated in this phase. All encounters occurred in the ED of the Mayo Clinic Hospital, Saint Marys Campus (Rochester, Minnesota), a quaternary care academic institution with approximately 77,000 annual ED visits. Appropriate parent/caregiver and clinician consent, approved by our institutional review board (IRB), was obtained before making these recordings. From these observations we used a predefined observation grid developed in previous studies^4^^,^^12–14^ to evaluate patterns of patient-parent conversations as well as nonverbal behaviors and attitudes. Additionally, we discerned strengths, challenges, patterns and unaddressed issues in current practice. These learnings were incorporated in the creation of prototype conversation aids.

### Prototyping and field testing

Field testing improves the understanding of the context in which the conversation aid will be used. In creating and iterating on prototypes, feedback was sought from the study team, clinicians, parents of children with acute otitis media, and patients from our ED patient advisory council. Stakeholders were asked to comment on the content, format, ease of use, how well it conveys current evidence, and to what extent the conversation aid brings to light context, values, and preferences in decision-making.

We piloted the conversation aid in five clinical encounters, each of which was followed by a brief interview with the parent and clinician. Five different pediatric emergency clinicians voluntarily participated in the field-testing. This process, which we have applied to several previous decision aids,^4^^,^^12–14^ was repeated until the study team deemed that we had a conversation aid that was appropriate for use in the ED. The suitability of the final prototype was not formally evaluated but yet decided based on consensus within the core study team after receiving informal feedback from clinicians and parents.

## RESULTS

### First prototype: paper-based folder

The first prototype (Supplementary Appendix S2) of the tool was modeled after our previously developed head injury and chest pain decision aids.^17^^,^^18^ This tool took the format of a paper-based folder that provided quantitative information about the duration of pain at 24 hours and 2 to 3 days with and without antibiotic treatment. Complications from antibiotics were also presented in numerical format. Through interviews, we found that parents preferred the terminology of “wait and see” as opposed to “wait and watch.” Similarly, parents did not like the term “side-effect,” so the terminology of “effects of illness and antibiotics” was utilized.

Issues that arose from the first prototype were mainly focused on improving the flow of the conversation aid. First, it was felt that there were too many words and that practitioners were reading more than interacting with parents. In addition, the presentation of pain at 24 hours and 2 to 3 days was “too busy,” had “too many dots” and “too many numbers to compare.” From this information, the consensus of the core team was to include only one timeframe for the comparison of pain. The decision was made to focus on pain at 2 to 3 days instead of 24 hours, because the longer time frame would more accurately reflect the path chosen. Also, additional feedback indicated a need for a greater visual distinction between antibiotics and no antibiotics. Through discussion with parents, the title of the conversation aid was changed from “Ear Infection Decision Aid” to “Ear Pain Decision Aid.” This change was made based on feedback from parents that the word “infection” might make parents more likely to want to use antibiotics. Additionally, we learned that the reason that most parents sought help was for ear pain rather than an ear infection. This was an important reframing of the conversation away from underlying medical causes toward the suffering that required addressing in the decision-making process.

### Second prototype: paper-based pocket card

Unlike the largely quantitative presentation used in the first version, the second prototype (Supplementary Appendix S3) sought to guide a conversation about the treatment of AOM. This took the form of cards that identified common concerns that arise in making ear pain treatment decisions and helped cultivate discussion between the practitioner and the parent on these issues.

The second prototype brought up concerns regarding being biased toward an antibiotic prescription, since the antibiotic section was placed on top. In addition, practitioners were concerned that having only two choices, “immediate antibiotics” or a “wait and see” prescription, was misleading as they wanted to have another option to “do nothing.” The decision was then made to change the options to: “wait and see,” “wait and see with prescription,” and “immediate antibiotics.” Other concerns brought up with the second prototype were that the dot pictograms were more difficult to compare since they were vertically and not horizontally stacked.

Practitioners requested a picture of the middle ear to show where the fluid builds up that causes pain in AOM. In addition, clinicians asked for quantitative information on the complication rates of AOM with and without antibiotics. Additional feedback was that the continued pain category in the pictograph was colored orange, the same as the ibuprofen/acetaminophen cup pictured above which brought up the concern that parents may confuse ibuprofen with continued pain.

### The third prototype: web-based electronic tool

**Figure 2.**
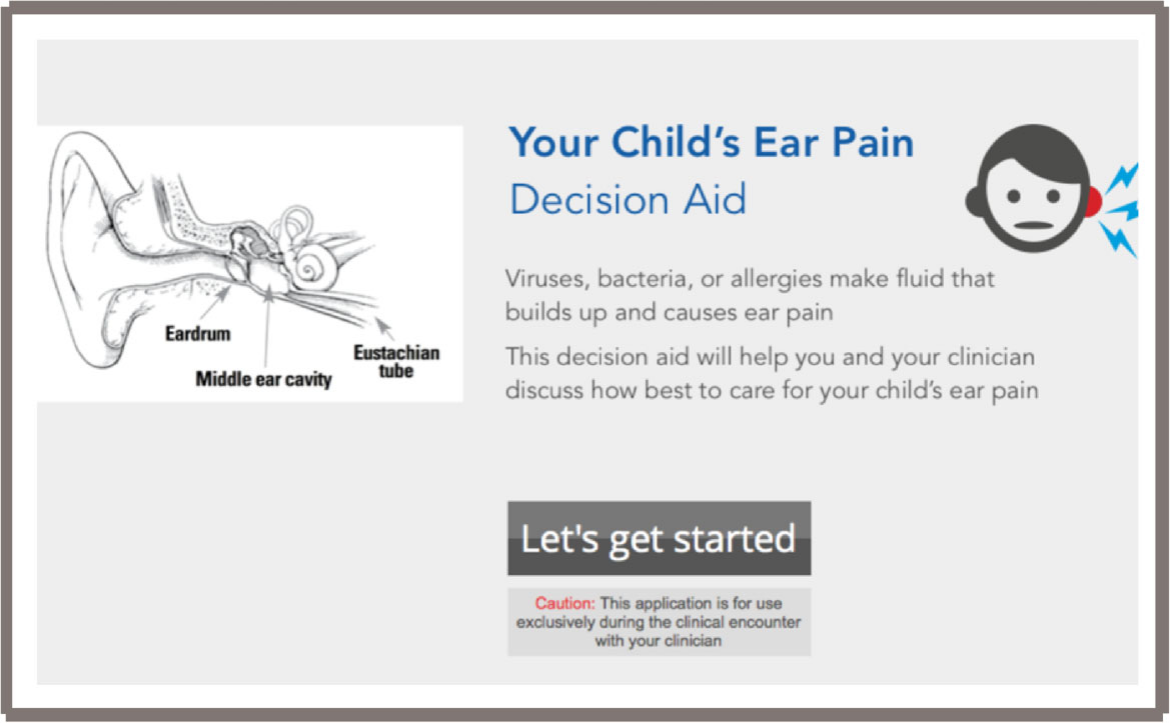
Web-based conversation aid, home page.

**Figure 3.**
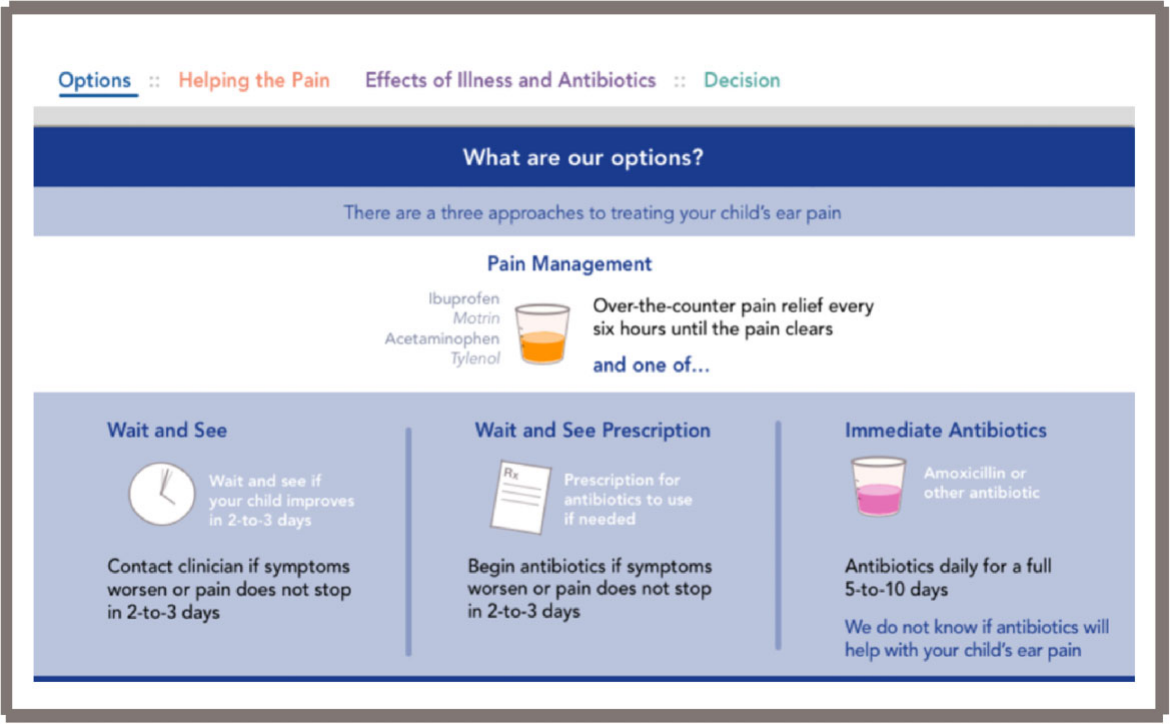
Web-based conversation aid, options page.

**Figure 4.**
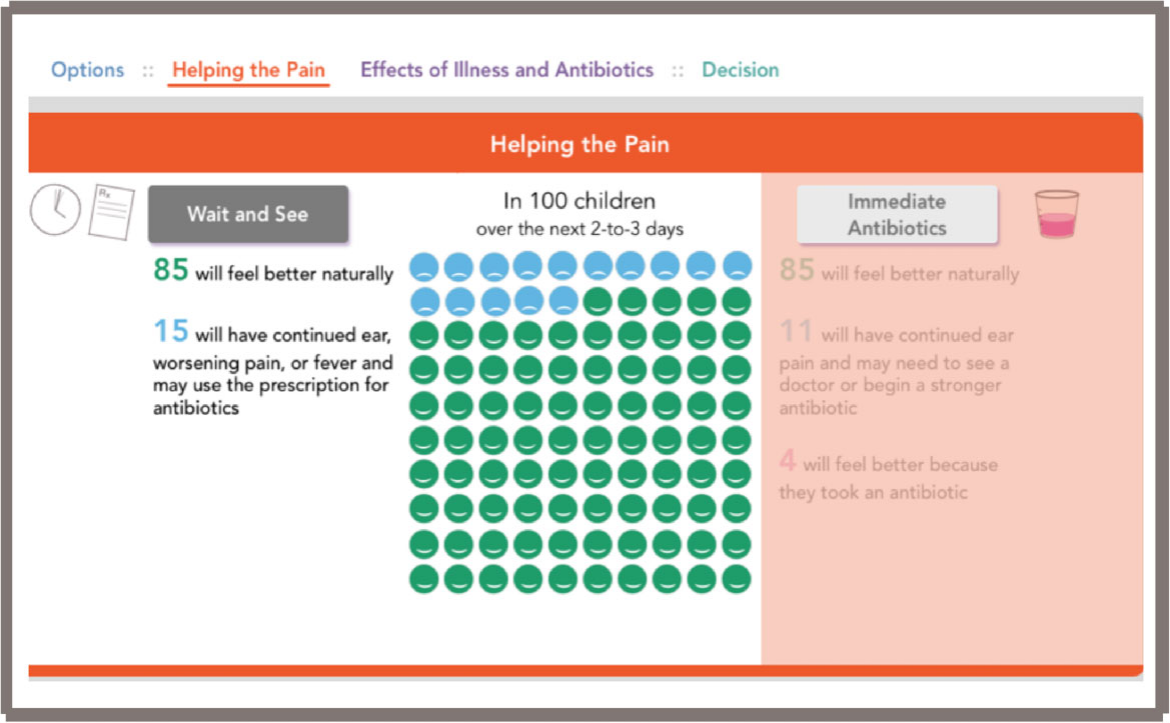
Web-based conversation aid, helping with pain page—toggled to “Wait and See.”

**Figure 5.**
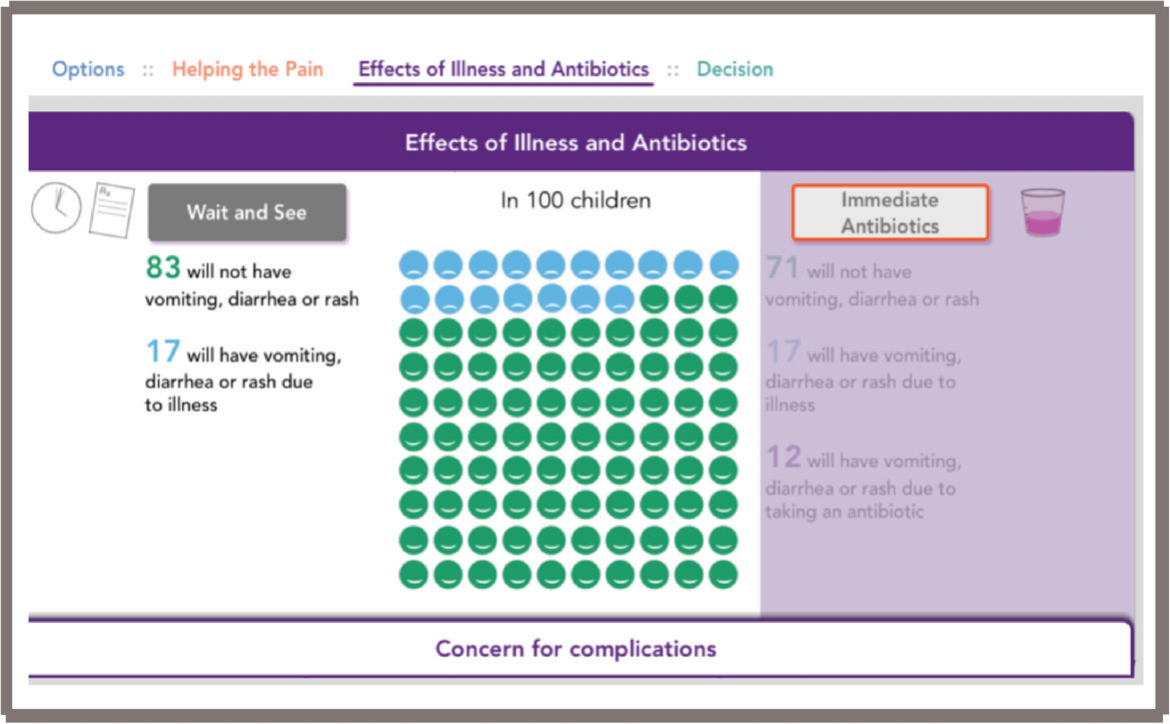
Web-based conversation aid, effects of illness and antibiotics page—toggled to “Wait and See.”

**Figure 6.**
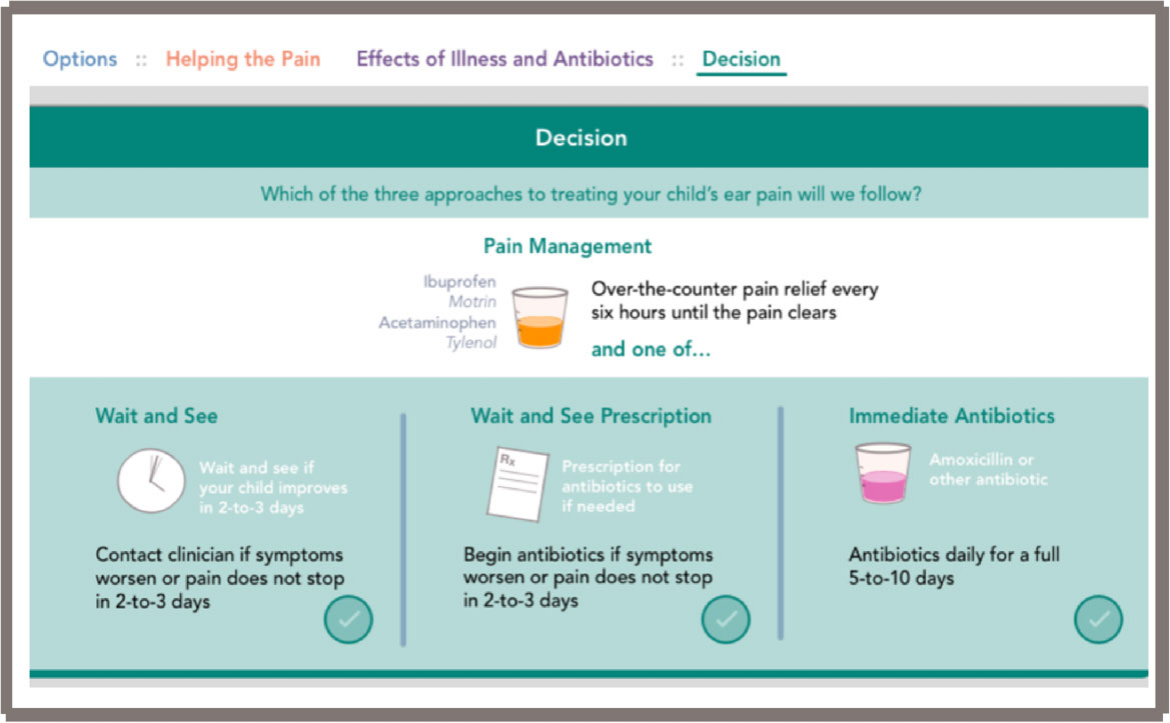
Web-based conversation aid, decision page.

After reviewing the first two tools and gathering informal feedback during prototyping and field-testing, by consensus of the core development team we decided to use the electronic format as the final version. This open access web-based tool is located at www.earpaindecisionaid.org (Figures 2–6). The decision aid starts with an opening page (Figure 2), and once “Let’s get started” is selected, it opens into the main field to discuss analgesia and the three options: “wait and see,” “wait and see with prescription,” and “immediate antibiotics” (Figure 3). Next, the practitioner should select next to the “options” area on “helping the pain” tab (Figure 4). This area allows the practitioner to toggle between the “wait and see” and “immediate antibiotics” on the impact of pain control and possible downstream effects. Next, in the header tabs “effects of illness and antibiotics” (Figure 5), the user can select between “wait and see” and “immediate antibiotics.” In addition, this section has a lower tab titled “concern for complications” that, when selected, brings up the rates of common complications of AOM. The rate of complications was not a significant concern of the parents surveyed; therefore, this information was embedded in case it is desired by the practitioner or parents. The final step of the conversation aid is the “decision” tab. When selected, analgesia is again reinforced, and the three options are presented for discussion between the parent and practitioner (Figure 6).

## DISCUSSION

Through a team-based iterative process, we incorporated the current evidence on AOM guided by parent and practitioner perspectives to develop the ear pain decision aid. It is a four-tab, web-based, conversation aid designed to facilitate an evidence-informed dialogue between the clinician and the parent in a busy acute care setting. The conversation aid presents three options regarding treatment of AOM, opening the discussion that not all ear infections need to be treated with antibiotics. In addition, the tool enables quick visual comparisons between immediate antibiotics and observation without antibiotics and the subsequent impact on pain, common effects of antibiotics and infection, and possible complications.

Parental input was vital to the development of the decision aid. From the initial parental interviews, the theme of pain control predominated. Many parents felt that as long as the child’s pain was under control they were more than willing to forgo antibiotics and actually welcomed not having the child on antibiotics. The data regarding pain control and the impact of antibiotic therapy is presented in a quantitative pictograph form. This quantitative approach appeals to some parents. Through parent interviews, it was also discovered that parents appreciated the time and building of trust that developed between them and the practitioner. Parents mentioned that even if they were “not into the numbers,” since the practitioner spent time explaining things they were more likely to follow through the agreed upon therapy and more likely not to use antibiotics.

While the conversation behind the management of non-severe AOM may start off by assuming that parents are inclined to the deliberate prescription of antibiotics, most surveyed parents had an appropriate understanding of its use in this context. A relatively high number of parents (57%) in the initial survey (Table 1) reported that antibiotics are only needed “sometimes”. This could represent the openness of parents for SDM in the setting of non-severe AOM, further highlighting the importance of having these evidence-informed conversations. However, given the small sample size, this finding may also represent a biased selection of parents with a high level of awareness and education regarding the use of antibiotics.

Practitioner input into the usability of the decision aid was also a key to the iterations. Though all three major versions of the conversation aid presented the same numerical data for pain, side effects, and complications, it was felt that the final design had the least degree of bias for or against antibiotics. Practitioners appreciated the anatomical drawing of the ear and middle ear on the title page to show parents and children how fluid can build up, causing pressure. Regarding usability, practitioners mentioned that it took two or three times through the decision aid to become comfortable with the flow and learn how to toggle between “immediate antibiotics” or “wait and see.” In addition, the “complications” tab, though initially cited as difficult to find, was pointed out by practitioners and parents to be appropriately placed in the lower aspect of the conversation aid as opposed to the main flow of the tool. Practitioners preferred the web-based tool over paper, even though having the website bookmarked took time, effort, and forethought. Having the conversation aid readily at hand on a computer was reported to outweigh the benefit of sending the parent home with the paper-based conversation aid.

## LIMITATIONS

There are several limitations that need to be acknowledged. First, despite the iterative creation of different prototypes, we have not formally compared the three options at the end of the process. Instead, we decided to choose the electronic format due to its relatively simplicity and because this version was optimized after incorporating all the feedbacks obtained during field-testing of the first two paper-based prototypes. Second, the tool was only field-tested with emergency physicians actively engaged in the development of the tool. Other specialists such as otolaryngologists, for example, were not involved in the development of the conversation aid. A more inclusive field-testing with outside clinicians could have led to a different final version. Third, the tool was developed using clinicians and parents of a single center in the USA, which may restrict its usability in other patient populations.

## CONCLUSIONS

Using a practice-based, human-centered approach, we developed the ear pain conversation aid which aims to facilitate dialogue between clinicians and parents of children diagnosed with non-severe AOM in the acute care setting. Its use as a tool for SDM is currently being evaluated in a randomized controlled trial (NCT02872558) in order to evaluate its impact on outcomes such as parent knowledge, antibiotic use, and complication rates.

## FUNDING

This study is part of the project entitled “Shared Decision Making in Parents of Children with Acute Otitis Media: The Acute Otitis Media Choice Trial,” and it was supported by a research grant of the Emergency Medicine Foundation through the category of “Patient-Centered Outcome Research.” JLA is the principal investigator of this research grant.

## AUTHOR CONTRIBUTIONS

JLA conceived the idea for the study, performed literature review, and led the development of the conversation aid. EPH was the senior author and mentor throughout the study. JPB and IGH designed and revised the conversation aid. LOJS performed methods review. All authors participated in writing this manuscript.

## SUPPLEMENTARY MATERIAL

Supplementary material is available at *Journal of the American Medical Informatics Association* online.

## Supplementary Material

ooab024_Supplementary_Data
