# Supplementary material for: Development of an electronic conversation aid to support shared decision making for children with acute otitis media
Source: JAMIA Open. 2021 Apr 19;4(2):ooab024. doi: 10.1093/jamiaopen/ooab024 (PMC8054029; doi:10.1093/jamiaopen/ooab024)
Supplement: ooab024_Supplementary_Data [file ooab024_supplementary_data.docx]

**SUPPLEMENTARY MATERIAL**

**Legend**

Appendix S1. Paper-based survey used in the developmental process of the Ear Pain decision aid.

Appendix S2. Prototype Version 1 (paper-based).

Appendix S3. Prototype Version 2 (paper-based, pocket-card format).

**Appendix S1.** Paper-based survey used in the developmental process of the Ear Pain decision aid.

Question 1. How old was your child when he/she was diagnosed with their most recent ear infection? Circle.

- < 6 months
- 6 months to 2 years
- 2 to 5 years
- 5 years

Question 2. How long ago was your child’s most recent ear infection? Circle.

- < 6 months
- 6 months to 1 year
- 1 to 2 years
- > 2 years

Question 3. How many ear infections has your child had in the past year? Circle.

- 1
- 2
- 3
- 4
- 5

Question 4. What symptom made you seek care for the ear infection? Circle all that apply and number from most important (1) to least important (8).

___ Fever

___ Pulling ear

___ Crying

___ Pain

___ Fussiness

___ Ear drainage

___ Difficulty Sleeping

___ Other

Question 5. What did you hope most to gain from your last visit for the ear infection? Number from most important (1) to least important (5).

___ Pain control

___ Reassurance

___A diagnosis

___ Antibiotics

___ Other

Question 6. What worried you most about your child’s last ear infection? Number from most important (1) to least important (5).

___ Fever

___ Infection spreading

___ Pain

___Difficulty sleeping

___ Ear drum rupturing

___ Other

Question 7. What was your child treated with? Circle all that were used.

- Tylenol or ibuprofen
- Antibiotics
- Ear drops
- Nothing
- I don’t remember

Question 8. How involved were you in the decision to treat your child? Circle.

- Extremely
- Very
- Moderately
- Slightly
- Not at all

Question 9. How long did it take for your child to feel better? Circle.

- 1 day
- 2-3 days
- 4-7 days
- > 1 week

Question 10. Circle the response that fits best your idea of treating ear infection.

*Antibiotics are needed to treat ear infections…:*

- Always
- Most of the times
- Sometimes
- Not very often
- Never
- Not sure

Question 11. Did your child have any problems after the ear infection was diagnosed? Circle all that apply.

- No problem
- Repeat ear infection within 2 weeks
- Mastoiditis
- Meningitis
- Cerebral venous thrombosis
- Other

Question 12. Which of these five statements best describes how you prefer to make medical decisions?

- I make decisions about my child’s health care
- I make decisions about my child’s health care after seriously considering my clinician’s opinion.
- My clinician and I share responsibility for making decisions about my child’s health care.
- My clinician makes decisions about my child’s health care, but seriously considers my opinion.
- My clinician makes decisions about my child’s health care.

**Appendix S2.** Prototype Version 1 (paper-based).

**Figure S2A**. Prototype Version 1 (paper-based), Part 1.

**Figure S2B**. Prototype Version 1 (paper-based), Part 2.

**Appendix S3.** Prototype Version 2 (paper-based, pocket-card format).

**Figure S3A.** Prototype Version 2 (paper-based, pocket-card format), Part 1.

**Figure S3B.** Prototype Version 2 (paper-based, pocket-card format), Part 2.

**Figure S3C.** Prototype Version 2 (paper-based, pocket-card format), Part 3.
